# Etanercept to Control Inflammation in the Treatment of Complicated Neurocysticercosis

**DOI:** 10.4269/ajtmh.18-0795

**Published:** 2019-01-02

**Authors:** Theodore E. Nash, JeanAnne M. Ware, Christina M. Coyle, Siddhartha Mahanty

**Affiliations:** 1 Laboratory of Parasitic Diseases, National Institutes of Allergy and Infectious Diseases, National Institutes of Health, Bethesda, Maryland;; 2 Department of Medicine, Albert Einstein College of Medicine, Bronx, New York

## Abstract

Manifestations of neurocysticercosis (NCC) are primarily due to host inflammatory responses directed at drug-damaged or naturally degenerating metacestodes (cysts) of the tapeworm *Taenia solium*. Prolonged high-dose corticosteroids are frequently required to control this inflammation in complicated disease, often causing severe side effects. Studies evaluating alternatives to corticosteroids are lacking. Here, we describe the clinical course of NCC in 16 patients prescribed etanercept (ETN), a tumor necrosis factor-alpha inhibitor to control inflammation resulting from anthelmintic treatment. Twelve patients with extraparenchymal NCC were administered ETN with corticosteroids (11/12, 91.7%) and/or methotrexate (9/12, 75.0%). The median age of the subgroup with extraparenchymal NCC was 40 years (range 26–57 years) and 66.7% were male. They were administered ETN for a median period of 311 days (range 31–461 days) and then followed for a median of 3.4 years (range 0.3–6.6 years). Among nine assessable patients, all improved clinically after starting ETN and one deteriorated transiently. Of the remaining three, one was lost to follow-up and two patients have improved but had not completed their assigned course. Four additional persons with recurrent perilesional edema (PE) episodes were given ETN for a median of 400.5 days (range 366–854 days) and followed post-ETN for a median of 1.7 years (range 0.2–2.4 years). All PE patients improved and two successfully tapered corticosteroids. Etanercept administration was associated with clinical improvement, stable disease, absence of recurrence, and lack of serious side effects. Etanercept appears to contribute to the control of inflammation and facilitate corticosteroid taper.

## INTRODUCTION

Neurocysticercosis (NCC) is an infection of the brain with the intermediate or cystic stage of the cestode parasite *Taenia solium*.^1^ Almost all the pathology and resulting disease is due to acute and chronic inflammation directed at degenerating cysts. The manifestations and severity of disease are modified by the number and location of cysts within the brain, the degree of degeneration, the presence and severity of associated inflammation, and cyst type. Specific clinical syndromes and pathologies reflect involvement of the major anatomic brain compartments. Seizures are the main clinical presentation of parenchymal cysts, usually associated with degenerating or calcified cysts with or without perilesional inflammation. Involvement of the ventricular system mostly gives rise to signs and symptoms due to the obstruction of cerebral spinal fluid (CSF) flow, ventriculitis, and periventricular inflammation, resulting in hydrocephalus. Racemose subarachnoid NCC (SUBNCC), a structurally and biologically aberrant form of the *T. solium* cyst stage, almost always involves the subarachnoid spaces of the brain, primarily the basilar cisterns and sylvian fissures. The morphology of SUBNCC is distinctive. It consists of varying amounts of proliferating parasite tissue components and vesicles usually in the absence of an identifiable scolex. The parasite’s continued growth leads to widespread central nervous system (CNS) involvement and life-threatening mass effects.^2,3^ Subarachnoid NCC is usually accompanied by varying degrees of cyst degeneration and inflammation, which occurs naturally or is initiated or worsened by cysticidal treatment. Communicating hydrocephalus, infarcts, focal nerve dysfunction, and localized brain atrophy are consequences of chronic inflammation.^1^ There are no randomized studies of the treatment of SUBNCC and no standardized treatment regimens, but most experts use prolonged cysticidal treatment along with high-dose corticosteroids.

Albendazole and praziquantel, singly or in combination, are the two cysticidal drugs available to treat NCC. Paradoxically, treatment provokes host inflammatory responses directed at the injured/dead parasites, resulting in disease deterioration.^4^ Although not well studied, there is a long experience using corticosteroids to control treatment-induced inflammation in multicystic or parenchymal NCC.^5^ Short-term use of corticosteroids is generally well tolerated. By contrast, long-term high-dose corticosteroid administration frequently results in severe and even life-threatening side effects.^6^

Even though their use in complicated NCC has never been formally studied, corticosteroids have become the standard treatment for suppression of inflammation in NCC. However, alternatives to corticosteroids are needed for a number of reasons. First, corticosteroids are absolutely contraindicated in certain situations, for instance when they cause psychosis. Second, corticosteroids may cause severe side effects, such as difficultly in controlling diabetes, cataracts, bone fractures, and avascular necrosis, which limits their utility, leading to interruption or premature cessation of therapy. Third, corticosteroids are sometimes ineffective or do not control disease manifestations at reasonable doses. Symptoms commonly develop when the corticosteroid dose is tapered and the addition of a second effective medication might result in complementary, added, or synergistic anti-inflammatory effects. By limiting or avoiding the need to raise the dose of corticosteroids, addition of another effective drug has a corticosteroid-sparing effect. Last, because the development of corticosteroid-induced side effects is directly related to the dose and amount taken, other medications that allow the use of reduced doses of corticosteroids throughout the course of treatment will necessarily lead to corticosteroid sparing and a decrease in side effects.

We have used methotrexate as a steroid-sparing/replacement medication for NCC for years.^7^ Although well tolerated, the use of methotrexate has several limitations. First, it has a delayed onset of action, so it cannot be used to quickly control inflammation. Second, at a usual dose of 20 mg/week, a safe and effective regimen commonly used to treatment rheumatoid arthritis (RA), it does not appear to be as effective in controlling acute inflammation in NCC compared with corticosteroids (T. Nash, personal communication). Last, it can cause liver injury, which can be confused with albendazole toxicity.

Tumor necrosis factor-alpha (TNF-α) is a pro-inflammatory cytokine that appears to play a pivotal role in the inflammatory host response in NCC.^8,9^ We hypothesized that inhibition of TNF-α with etanercept (ETN), an anti–TNF-α biological agent that is routinely used in the treatment of RA, psoriasis, and ankylosing spondylitis, might be useful in the control of inflammation in NCC. Although it does not normally cross the blood–brain barrier (BBB) into the CNS,^10^ one of the hallmarks of NCC is BBB dysfunction and leakage of blood constituents into the CSF and brain. Anti–TNF-α agents are effective in the treatment of other CNS diseases,^11,12^ such as neurosarcoidosis, likely because of a leaky BBB. In addition, a recent study by our group demonstrated that ETN profoundly lowered pro-inflammatory cytokines including TNF-α in the brain of *T. solium*–infected pigs following praziquantel treatment.^9^ These profound inhibitory effects indicate that ETN was able to cross into the brain.

Here, we present the clinical course of a subgroup of patients with NCC treated with ETN. We found that almost all the recipients demonstrated clinical improvement on treatment with minimal side effects.

## METHODS

This is an observational study of a subset of patients with proven NCC^13^ enrolled in the Institutional Review Board-approved protocol 85-I-0127 of the Laboratory of Parasitic Diseases, National Institute of Allergy and Infectious Diseases in the National Institutes of Health (NIH). This protocol allows the evaluation, treatment (including the use of the novel immunosuppressive agents methotrexate and ETN), and follow-up of patients with NCC. All patients signed the protocol informed consent. These patients were extensively evaluated and followed, including an initial unenhanced computed tomography scan of the brain and serial magnetic resonance imaging (MRI) of the brain and/or spine, when indicated. Evaluation of enrolled participants included a Western blot test for cysticercosis and *Strongyloides stercoralis* serology, both performed by the Centers for Disease Control and Prevention, and QuantiFERON-TB Gold assay (QFT-G; Cellestis Limited, Victoria, Australia) for latent tuberculosis (TB). The manufacturer of ETN, which is U.S. Food and Drug Administration approved for treatment of RA, psoriatic arthritis, ankylosing spondylitis, and plaque psoriasis, had no knowledge of or involvement in our use of the drug in NCC. Most patients were followed up frequently in clinic, often weekly or biweekly following initiation of treatment and then less often, roughly every month while under active treatment and undergoing a corticosteroid taper. The frequency of follow-up visits varied in the early follow-up period, but after the patients were clinically stable off of all drugs and without relapse, they were followed up on a semi-annual or annual basis indefinitely.

Cysticidal treatment consisted of either albendazole (15 mg/kg/day) or praziquantel (50–100 mg/kg/day) or combined treatment with both agents. The range of the starting dose of dexamethasone per day was routinely between 10 and 16 mg/day, with a typical tapering regimen, beginning shortly after clinical stabilization, of roughly 1 mg/week decrease until about 3 mg/day dexamethasone and then switched to approximately 20 mg prednisone and tapered at 1 mg/day prednisone/week. Patients were also routinely prescribed Bactrim to prevent *Pneumocystis* pneumonia, a protein pump inhibitor to prevent gastritis and bleeding, and other drugs as medically indicated. Recurrence of symptoms during the taper usually required a transient increase in corticosteroid dose followed by resumption of tapering at the higher dose. Failure to control symptoms during the corticosteroid taper was one of the reasons for initiating ETN. If cysticidal treatment was continued, the lowest dose of corticosteroids controlling enhancement and symptoms was maintained. A course of isoniazid was given if latent TB was documented, as recommended by the American Thoracic Society guidelines, along with or before starting ETN, depending on the perceived need for ETN. Lumbar punctures for CSF were not consistently performed initially, but in patients enrolled more recently, serial lumbar CSF evaluations were frequently performed to assess the usefulness of CSF parameters as valid end points of therapy for SUBNCC.

Indications for the administration of ETN fell under two main categories. First, it replaced corticosteroids when their use was contraindicated, for instance in patients who had a history of or developed steroid-induced psychosis (two patients in our series) or in patients with repeated perilesional edema (PE) episodes resulting in uncontrolled symptoms or seizures. Because of the propensity for a corticosteroid taper to exacerbate PE around^14^ calcifications already experiencing edema and induce newly formed PE around calcifications that did not previously have any,^15^ we avoided using corticosteroids to control symptoms caused by PE. The second common reason was for control of inflammation when symptoms occurred during a patient’s corticosteroid taper. The addition of a second potentially effective drug increased immunosuppression and thereby facilitated corticosteroid sparing by allowing for successful corticosteroid taper and corticosteroid replacement when longer term control of inflammation was required. Half the normal dose of ETN (25 mg/week) used in other diseases was used in the patients treated earlier (10/16) because of safety concerns. The dose was increased to 50 mg/week in patients seen later (7/16, one person given both doses). It was continued for varying durations depending on the clinical response of the patient. Courses of ETN were administered multiple times to some individuals for specific or recurring symptoms separated by weeks to months. A period of time of continuous administration, excepting temporary missed dosing, is defined as a single course.

Cestode antigen assays were performed using either a Luminex-based bead array assay (T. Nutman, unpublished data) using reagents supplied by Dr. Dorny (Department of Biomedical Sciences, Prince Leopold Institute of Tropical Medicine, Antwerp, Belgium) or a cysticercosis antigen ELISA commercial kit (ApDia, Turnhout, Belgium). Standard curves generated by using *T. solium* antigen allowed the conversion of optical density to antigen concentration of ng/mL and comparison between assays run on different days. The sensitivity of both assays was < 3 ng/mL and they were equally able to discern undetectable from very low levels, which was interpreted as a measure of successful treatment and a rationale to stop cysticidal drugs.

### Illustrative case 1.

A 51-year-old Hispanic male presented in 2009 with a giant left sylvian cyst and extensive SUBNCC involving most of the basilar cisterns and right sylvian fissure. The giant cyst was removed and he was started on albendazole and corticosteroids. Nevertheless, his disease progressed to massive involvement of the basilar cisterns and hydrocephalus. Retreatments over the next 3 years resulted in periods of regression followed by progression due to poor compliance because he could not afford the medication. He again came to medical attention in April 2016 after being lost to follow-up for 4 years. He was unable to walk, was severely demented, and was unable to care for himself. He complained of headaches and decreased vision in the right > left, and reported sounds in his left ear. Magnetic resonance imaging revealed massive compensated hydrocephalus and less extensive SUBNCC. He was restarted on albendazole, praziquantel, and dexamethasone. He was first seen at NIH in August 2016. He was able to ambulate and was disoriented to the date, place, and name of the president. He still required constant care and complained of vision loss and hearing sounds. These symptoms could be attributed to optic atrophy and 5th cranial nerve involvement with the disease. Magnetic resonance imaging showed massive compensated hydrocephalus, less spinal disease, and improved but some residual basilar SUBNCC. Lumbar punctures revealed white blood cell count (WBC) of 34 mm^3^, 66% lymph, and 34% other cells, including 26% eosinophils, protein 83 mg/dL, and glucose 43 mg/dL (serum glucose 263 mg/dL). He was restarted on high-dose corticosteroids, albendazole, and praziquantel with worsening diabetes and acute corticosteroid-induced psychosis manifested as mania. Corticosteroids were stopped and he was begun on 50 mg ETN subcutaneously as his only immunosuppressive medication. He improved markedly and became oriented to time and place, was able to take care of himself, and worked doing unskilled labor. Etanercept was stopped in November 2017 and albendazole and praziquantel on March 2018, after 28.5 months on ETN. At that time, a CSF examination showed improved inflammatory parameters with WBC 9 cells/mm^3^, protein 58 mg/dL, and glucose 52 mg/dL (serum glucose 126 mg/dL). The cestode antigen decreased from 157 ng/mL to a barely detectable concentration of 2 ng/mL. The patient had no symptoms on cysticidal treatment with ETN or on ETN alone. He has continued to do well to the present.

### Illustrative case 2.

A 57-year-old male originally from Puerto Rico was first diagnosed with NCC in October 2005 after a grand mal seizure due to degenerating parenchymal and viable NCC cysts. As part of his work, he had traveled extensively to endemic regions. The cysts calcified after treatment with albendazole and corticosteroids, but he developed repeated episodes of PE involving left frontal and right parietal calcifications. Except for a single PE episode in 2011 involving the right parietal lesion, manifested as a seizure, the patient was stable until April 7, 2014, when he had a generalized seizure accompanied by a PE episode involving a left frontal calcification. He had two more generalized seizures on May 20, 2014, and September 4, 2014, associated with continued PE of the left frontal calcification despite increases in anti-seizure medications. He was started on a brief course of corticosteroids in an attempt to decrease seizures, but when his dose was tapered from 60 mg/day prednisone to 5 mg/day on September 28, 2014, he experienced another grand mal seizure and developed a new PE episode involving the previously uninvolved right parietal calcification. The left frontal PE persisted. Prednisone was increased to 20 mg/day and he was referred to NIH on October 6, 2014. Besides seizures, his major complaint was headaches. He was started on 25 mg/week etanercept and a slow corticosteroid taper from 20 mg prednisone/day, methotrexate 20 mg/week, folic acid, and maximization of anti-seizure medication. After corticosteroids were discontinued on July 8, 2014, while maintaining treatment with ETN and methotrexate, his headaches improved dramatically. He reported that he was 90% better. He had prior skin lesions eventually diagnosed as granuloma annulare that continued to enlarge. Because of a concern that enlargement of these was worsened by ETN, the drug was stopped on October 8, 2015; methotrexate was continued until May 20, 2016. About 2 weeks after stopping ETN, he again began to suffer from severe headaches without seizures. There was a possible episode of asymptomatic PE demonstrating a questionable wisp of edema involving the right parietal calcification. Although all systemic immunosuppressive medications were stopped, the granuloma annulare lesions continued to enlarge despite local corticosteroid treatment. He has been clinically stable without seizures or PE on unchanged anti-seizure medications but with headaches. Because headaches returned after stopping ETN while on methotrexate, a benefit of ETN is suggested.

### Illustrative case 3.

After migrating to the United States from India 18 years before diagnosis, the patient developed signs and symptoms of spinal arachnoiditis. Despite extensive evaluations at a number of facilities, including several internationally recognized medical institutions, and a diagnostic biopsy, she remained undiagnosed for 15 years until the Fall of 2017. She had recently been started on anakinra, a recombinant human interleukin 1 receptor antagonist to control inflammation due to a process of unclear cause. She was able to tolerate a taper of prednisone from 35 mg/day to 29 mg/day associated with subjective improvement in symptoms. But despite multiple attempts, she was unable to reduce the dose of prednisone any lower. Following the diagnosis of NCC, she was started on ETN, 50 mg/day, and albendazole and praziquantel therapy; anakinra was continued because of the patient’s insistence that it had controlled some of her symptoms. After being placed on ETN, her activity level increased and she continues to gradually lower her daily dose of prednisone below the previously achievable minimum dose (29 mg/day) to 0.5 mg/day at present.

## RESULTS

Sixteen persons were administered one or more courses of ETN (Table 1). Among these, 12 persons (75.0%) had extraparenchymal disease, 11 (68.8%) persons had SUBNCC, and one (6.3%) had ventricular disease due to a retained 4th ventricular cyst. Another four patients (25%) experienced repeated incapacitating PE with seizures (Tables 1 and 2).

**Table 1:** Clinical characteristics, treatment, and follow-up of all study participants

Number	16	–
Age (median, years)	40 (range 19–57)	NA
Gender (% male)	10/16 (62.5%)	NA
Years of follow-up (median)	3.8 (range 0.2–8)	NA
Assessable	13/16 (81.3%)	NA
Type of neurocysticercosis	SUBNCC	11/16 (68.8%)
	Perilesional edema (calcific parenchymal)	4/16 (25.0%)
	Ventricular	1/16 (6.3%)
Median total days administered (range)	361 (range 41–854)	–
Number of courses (persons)	Single course	12 total (10 completed courses in 10 persons and two ongoing courses in two persons)
	Multiple courses	2,3,2,2 courses in each of four persons
	Total courses in persons	21 courses in 16 persons (19 courses completed, two on-going courses)
Dose of ETN	25 mg/week	11 courses
	50 mg/week	10 courses
Time followed post-ETN (median, years)	–	2.5 years (*n* = 14, range 0.2–5.6 years)
Other immunosuppressive drugs	Corticosteroids	12/16 (75.0%)
	Methotrexate	10/16 (62.5%)
	Other	1/16 (6.3%)
	ETN alone	3/16 (18.8%)
Persons on concomitant cysticidal drug(s)	–	10/16 (62.5%)

ETN = etanercept; NA = not applicable.

**Table 2:** Overview of perilesional patients

Patient number	Age (years)	Gender	Followed at National Institutes of Health (year)	Duration etanercept (days)	Duration followed up post-etanercept (year)	Albendazole and/or praziquantel	NCC disease type	Medication regimen	Outcome
1	57	M	3.4	366	2.4	None	Calcifications with multiple PE episodes	Corticosteroids to corticosteroids + 25 mg etanercept to 25 mg etanercept	No seizures or PE on etanercept after taper, headaches reoccurred after stopping etanercept
3	29	F	6.8	419	2.5	None	Calcifications with multiple PE, lateral ventricular entrapment, possible old subarachnoid NCC	50 mg etanercept	Headaches stopped and no PE on etanercept, PE returned after stopping
5	19	F	3.5	382	0.9	None	Calcification with multiple PE, treated multiple times with etanercept	50 etanercept	Headaches improved on etanercept and returned on stopping. No further PE
16	47	M	2.6	854	0.2	None	Calcification with multiple PE involving the motor strip on the right brain controlling left tongue and the left side of the face	Corticosteroids + methotrexate to corticosteroids + 25 mg etanercept to 25 mg etanercept	One further PE, less seizures, corticosteroid taper successful. Regained ability to eat and drink without provoking seizures. Overall improved with decreased focal seizures

NCC = neurocysticercosis; PE = perilesional edema episodes.

Most of the patients who received ETN had complicated SUBNCC. A brief summary of their demographics and treatment is shown in Table 3, and a more detailed summary, the reason for administration of etanercept, and outcome of treatment are provided in Supplemental Table 4. The median age of the extraparenchymal subgroup was 40 years (range 26–57 years) and 66.7% were male. They were followed up at NIH for a median of 4.4 years (range 0.7–8 years), administered ETN for a median period of 311 days (range 31–461 days), and then followed up for a median of 3.4 years (range 0.3–6.6 years). Etanercept was administered when symptoms and/or signs developed during a corticosteroid taper in 58.3% (7/12) of patients to enhance control of inflammation by adding immunosuppressive activity and also achieving some degree of corticosteroid sparing and replacement in most. Etanercept was added to specifically replace corticosteroids in one patient who developed corticosteroid psychosis; in two others, ETN was used with the intent of replacing corticosteroids as the primary anti-inflammatory agent. Seven patients (7/12, 58.3%) were also given methotrexate in addition to ETN. In all but one assessable patient, methotrexate was stopped before ETN, which was then continued as the sole anti-inflammatory agent for varying durations of time.

**Table 3:** Overview of extraparenchymal patients

Patient number	Age (years)	Gender	Years followed at NIH	Duration of etanercept (days)	Followed up post-etanercept (year)	Etanercept dose (mg/week)	Use of cysticidal medication(s) with etanercept	Disease type	Reason for etanercept	Medication regimens	Outcome
2	35	M	4.1	31	0.0	50	Yes	SUBNCC	Failed corticosteroid taper, added immunosuppression and sparing	Corticosteroids **+** methotrexate to corticosteroids **+** methotrexate **+** 50 mg etanercept	Short-term improvement. lost to follow-up at 31 days
4	41	F	0.4	NA	NA	50	Yes	Spinal SUBNCC	Failed corticosteroid taper + anakinra, added immunosuppression and corticosteroid sparing	Corticosteroids + anakinra to corticosteroids + 50 mg etanercept + anakinra	Tapered from 29 mg prednisone to 0.5 mg with mild improvement of symptoms due to spinal disease. Continues on etanercept
6	41	F	5.0	41	4.9	25	Yes	SUBNCC	Added immuosuppression, corticosteroid sparing	Corticosteroids + methotrexate + 25 mg etanrecept to corticosteroids	Improved short term but on taper developed neurological symptoms controlled on high-dose corticosteroids alone
7	51	M	1.6	380	0.3	50	Yes	SUBNCC	Corticosteroid replacement for corticosteroid psychosis	50 mg etanercept	Dramatic clinical improvement
8	57	M	0.7	NA	NA	50	Yes	SUBNCC, spinal	Added immunosuppression, corticosteroid replacement, and sparing	Corticosteroids to corticosteroids + 50 etanercept	Still on etanercept, moderate clinical improvement of pain due to spine involvement
9	41	F	4.0	699	2.1	25,50	Yes	SUBNCC, ventricular	Failed corticosteroid taper, added immunosuppression and sparing	Corticosteroids to corticosteroids + methotrexate to corticosteroids + methotrexate + 50 mg etanercept to 50 mg etanercept	Taper successful after the dose of etanercept was increased and did well on etanercept alone
10	30	M	3.6	272	2.6	25	Yes	SUBNCC, calcification	Failed corticosteroid taper, added immunosuppression and sparing	Corticosteroids to corticosteroids + 25 mg etanercept	Taper successful after the dose of etanercept was increased and did well on etanercept alone
11	37	M	6.6	229	5.6	25	Yes	SUBNCC, calcifications	Failed corticosteroid taper + methotrexate, added immunosuppression and sparing	Corticosteroids + methotrexate to 25 mg etanrecept + corticosteroids + methotrexate + 25 mg etanercept to methotrexate + 25 mg etanercept	Taper successful and avoided worsening in avascular necrosis on corticosteroid side effects
12	26	M	8.0	461	5.6	25	Yes	SUBNCC	Failed corticosteroid taper, added immunosuppression and sparing	Corticosteroids to corticosteroids + methotrexate to corticosteroids + methotrexate + 25 mg etanercept + methotrexate to 25 mg etanercept + corticosteroids to 25 mg etanercept	Taper successful and avoided further worsening of avascular necrosis of the hip
13	41	M	4.9	350	3.2	25	Yes	SUBNCC	Added immunosuppression	Corticosteroids to 25 mg etanercept corticosteroids + methotrexate to 25 mg etanercept to methotrexate to methotrexate	Developed large vessel stroke after completing therapy, resulting in retreatment that avoided additional vascular complications
14	28	M	6.3	356	4.6	25	No	Ventricular calcifications	Added immunosuppression and corticosteroid sparing	Corticosteroids to corticosteroids + methotrexate + 25 mg etanercept to corticosteroid + 25 mg etanercept to 25 mg etanercept	Taper successful and on prolonged etanercept with loss of priventricular edema and associated symptoms
15	39	F	4.7	210	3.4	25	No	SUBNCC	Failed corticosteroid taper, added immunosuppression, and replacement	Corticosteroids + methotrexate to 25 mg etanercept + corticosteroids + methotrexate + 25 mg etanercept to methotrexate	Taper successful with improved symptoms and loss of corticosteroid side effects, although improved still has headaches and depression, but no further transient episodes of hemiparalysis

NIH = National Institutes of Health; SUBNCC = subarachnoid neurocysticercosis.

Three persons in the extraparenchymal subgroup are not assessable. One patient was lost to follow-up while on ETN on day 31 (patient 2). Although there was early improvement, longer term assessment was not possible. The two other nonassessable persons (patients 4 and 8) have improved on ETN (Table 3) but await final assessment after ETN is stopped. Of the remaining nine patients, all but one clinically improved after the initiation of ETN. The patient who worsened after starting ETN had extensive SUBNCC and a complicated course both before enrollment and following initiation of treatment at NIH. She had been recently started on treatment including cysticidal drugs, ETN, methotrexate, and dexamethasone. About 6 weeks later she clinically deteriorated for unclear reasons, resulting in a modification and simplification of her treatment regimen that did not include ETN. Although the patient did clinically well after all treatment was stopped, 1 year later she had an asymptomatic recurrence of subarachnoid disease requiring retreatment. Two years after finishing her second course of treatment, she is clinically well. Currently, all assessable patients are stable, improved clinically without recurrence on follow-up after cessation of drugs for NCC treatment. Other than the one patient in whom ETN was stopped, ETN appeared safe. No flare of brain inflammation occurred and no specific side effects or serious adverse events that could be attributable to ETN, including infections or increased difficulty affecting cure were observed. In one person, erythema annulare worsened while on ETN and methotrexate but continued to enlarge 17 months after stopping ETN. Subsequently, methotrexate was also stopped but his skin lesions continued to enlarge despite treatment with topical corticosteroids. Therefore, the development and enlarging erythema annulare lesions are unlikely related to immunosuppression due to ETN or methotrexate.

Four persons were given ETN after they developed repeated incapacitating episodes of PE around calcified cysts. A summary of their details of treatment is presented in Table 2 and an expanded summary in Supplemental Table 4. They were followed up at NIH for a median of 3.5 years (range 2.6–6.8 years), administered ETN for a median of 401 days (range 366–854 days), and followed up posttreatment with ETN for a median of 1.7 years (range 0.2–2.4 years). Two referred patients had been prescribed corticosteroids to control PE and symptoms, but both developed recurrent seizures during the corticosteroid taper; in one patient, a second noninvolved calcification developed PE (patient 1) in addition to the chronic persistence of PE around the left frontal calcification. This patient improved after tapering corticosteroids on ETN and methotrexate with no further seizures due to a dramatic decrease in headaches. However, on the concern that granuloma annulare lesions had worsened on ETN, it was stopped while methotrexate was maintained. In this patient, headaches resumed 2 weeks later, but without recurrent seizures. Patient 16 had repeated PE episodes causing generalized and focal seizures involving the left tongue, face, and left side of the body. These were partially controlled with high-dose corticosteroids, but recurred with attempts to taper them. He showed staggered improvement after starting ETN. About 1 month after starting ETN, he clinically improved but experienced another episode of PE with a seizure, which was initially interpreted as a treatment failure. Our intention was to stop ETN and continue corticosteroids and perhaps use another anti-inflammatory agent. However, ETN was continued because on reevaluation the patient related that he had significantly improved after starting ETN; the disconcerting dysesthesia on the left side of the tongue was much improved. Seizure activity resolved and the patient gained increased ability to eat and drink without provoking focal seizures. The patient tapered off corticosteroids and eventually stopped ETN. He had one further episode of seizures in the absence of PE after he ran out of seizure medication. Patient 5 experienced unrelenting headaches and visual symptoms due to a right occipital calcification, undergoing multiple symptomatic episodes of PE; her unbearable headaches and visual symptoms remained controlled when given ETN but worsened when ETN was stopped. After addition of ETN, no further PE episodes occurred. Patient 3 sustained multiple PE episodes involving multiple calcifications, manifested as headaches, irritability, and personality changes. She improved dramatically on treatment with ETN but experienced another PE episode involving multiple calcifications after stopping the drug. Etanercept was restarted, but the patient stopped ETN after she misinterpreted signs and symptoms from a cervical disc as due to ETN. She is stable on anti-seizure medication. Overall, all the patients clinically improved on ETN but one patient had an episode of PE a month after starting treatment.

## DISCUSSION

Most of the pathology and clinical manifestations of NCC can be directly or indirectly attributed to the host inflammatory responses directed against cysts and cyst antigens.^1^ Although viable cysts are associated with little or no inflammation, degenerating cysts that develop as a consequence of the natural progression of disease or following cysticidal treatment incite acute and then chronic inflammation that leads to considerable morbidity and mortality. Shortly after praziquantel was first used to treat NCC, the need to suppress treatment-induced inflammation was recognized and, later, treatment was modified to suppress inflammation arising from the natural course of disease.^4^ Although its use is now almost universally accepted, the empirical use of corticosteroids in complicated NCC has neither been subjected to randomized controlled trials nor standardized.^5^ There is one randomized study comparing higher with lower dosing of corticosteroids in multicystic parenchymal NCC.^16^ Consequently, the type, dose, and duration of corticosteroid treatment vary and are dependent on the philosophy and experience of the treating physicians. The lack of randomized studies and recognized standard regimen in the treatment of SUBNCC disease leads to wide variability in the use and dosing of corticosteroids.

Despite the lack of definitive studies, prolonged high doses of corticosteroids are commonly used in SUBNCC (as well as in other forms of NCC) to moderate treatment-related side effects induced by cysticidal treatment and inflammation that occurs as part of the natural disease process.^17^ High-dose long-term corticosteroid treatment commonly causes severe and even life-threatening side effects that necessitate measures to limit toxicity. Replacement medications to achieve corticosteroid sparing are commonly used to limit corticosteroid side effects in other diseases. Unfortunately, there is little experience with other anti-inflammatory agents in NCC,^7^ and there are no randomized controlled trials to provide evidence of the clinical benefit of alternative agents in complicated NCC.

Our rationale for using TNF-α blockade is based on the key role attributed to this pro-inflammatory cytokine in inflammation involving the CNS in many diseases, including NCC. Tumor necrosis factor-alpha is an important modulator of inflammation in experimental models of inflammatory conditions of the brain in humans.^18–22^ In these studies, ETN decreases inflammation and controls the underlying disease. In investigations of the role of TNF-α in NCC, the level of TNF-α is increased in the CSF of patients with severe SUBNCC disease in some studies,^8,23^ but in others the role of TNF is less certain or not possible to resolve.^24^ In a naturally infected pig treatment model of parenchymal NCC, the expression of TNF-α mRNA in the inflammatory cells associated with the capsules of cysts with BBB leakage following treatment with praziquantel was 140 times greater than that in corresponding capsules of cysts without BBB leakage. Increases in other pro-inflammatory cytokines were also observed.^25^ In a later study using the same treatment schedule and methods, infected pigs pretreated with ETN demonstrated a profound inhibition of the dramatic increase in TNF-α and other pro-inflammatory cytokines seen posttreatment.^9^ These data implicate TNF-α as a major contributor to the acute inflammation that occurs in humans and pigs within the first week of cysticidal treatment.

Etanercept is a recombinant molecule consisting of TNF receptor 2 fused to the Fc fragment of the human immunoglobulin IgG1. This protein binds free TNF-α, competitively inhibiting its binding with the corresponding receptor. It has been used extensively in the treatment of RA and other inflammatory conditions.^26,27^ In RA, disease symptoms and joint pathology are significantly better when treatment with ETN is combined with methotrexate compared with either drug alone.^28^ Because a number of patients received both drugs, it is possible that clinical improvement could have been due to both drugs combined. Etanercept has relatively few significant side effects, particularly when compared with high-dose corticosteroids. Although ETN does not cross the BBB in the normal brain,^10,29^ the BBB is regularly compromised in NCC and even more so in SUBNCC, as demonstrated by the increase in gadolinium enhancement with MRI. Extensive enhancement and extravasation of Evan’s blue into capsules of brain cysts in *T. solium*–treated pigs amply prove that normally excluded molecules enter the brain in NCC.^25^ Other anti–TNF-α monoclonal antibodies, used in the treatment of patients with other inflammatory conditions such as neurosarcoidosis,^11,12^ are able to enter the CSF likely due to compromised BBB. Furthermore, as noted in the study in pigs, ETN pretreatment in vivo prevented the expected increase in TNF-α seen in the brain of naturally infected, experimentally treated pigs with NCC. This result can only be explained by the entry of ETN into the brain parenchyma.^9^

Etanercept was started in each subject not as part of a preconceived study but because of a clinical need that arose during treatment of individual patients, primarily to limit corticosteroid use and/or as an attempt to better suppress ongoing brain inflammation. The most common clinical situation was control of inflammation in SUBNCC disease and the second most common reason was to control inflammation and symptoms associated with PE episodes around calcifications. One patient with persistent PE due to a retained 4th ventricular cyst also received ETN. The use of corticosteroids to treat perilesional episodes is problematic because of the possibility of developing worsening rebound edema around involved calcifications or the induction of inflammation and edema around previously noninvolved calcifications during the corticosteroid taper.^15^

As a consequence of the varied clinical situations, the reason for starting ETN, the timing of its use, and the dose and duration differed among patients. Therefore, assessment of benefit is based on each patient’s response after initiation of ETN relative to their clinical status pretreatment. These included the ability to taper corticosteroids without developing new or worsening symptoms, improvement of symptoms, worsening temporally related to stopping ETN, an increase or decrease in drug side effects, and failure to cure or increase in recurrent disease. In most situations, the apparent benefit of the addition of ETN was multifold with some degree of additive immunosuppression, corticosteroid sparing, and replacement. A typical scenario was the addition of ETN to control symptoms that developed during the process of tapering corticosteroids. The addition of ETN in many instances allowed control of symptoms without needing to substantially increase the dose of corticosteroids. At the end of treatment, all our patients with SUBNCC were clinically improved, stable, and without relapse. Of the total 13 assessable patients, all but one experienced clinical improvement temporally related to start of the drug.

There are some disadvantages using ETN in this population. First, the drug is expensive, particularly in comparison with corticosteroids. Second, it requires subcutaneous injections. Third, similar to patients treated with high-dose corticosteroids, there is increased susceptibility to reactivation or new infection with TB, fungal infections (such as histoplasmosis), and susceptibility to other systemic infections, as well as other side effects, although these infections did not occur in our cohort.

Our experience using ETN is anecdotal and needs to be confirmed. As is common in other patients with complicated diseases, many were ill and, therefore, treated concurrently for a number of conditions or complications. They were commonly given multiple immunosuppressive medications, so attributing clinical improvement to ETN alone is not always possible. Nevertheless, we observed a persistent association of ETN treatment with clinical improvement and, taken together with its acceptable safety profile, suggest its utility as a corticosteroid-sparing/replacement medication. Randomized control trials of ETN are warranted to evaluate and establish efficacy.

## Supplementary Files

Supplemental tables
