# Supplementary material for: Etanercept to Control Inflammation in the Treatment of Complicated Neurocysticercosis
Source: Am J Trop Med Hyg. 2019 Jan 2;100(3):609–16. doi: 10.4269/ajtmh.18-0795 (PMC6402894; doi:10.4269/ajtmh.18-0795)
Supplement: Supplementary file 1 [file tpmd180795.SD1.pdf]

The following are supplemental materials and will be published online only

Table 4. Expanded summary of rationale for treatment and outcome of illness

Patient number	Expanded summary of course of illness
1	The patient presented with seizures due to a degenerating cyst with documented viable cysts that calcified after cysticidal treatment. Referral to the NIH was due to multiple episodes of PE that were not controlled on anti-seizure medications and reoccurred and/or initiated new PE upon attempts at lowering or stopping corticosteroids. He was first evaluated at NIH in the midst of a series of PE episodes provoked by a fast corticosteroid taper. ETN and methotrexate was started allowing a successful corticosteroid taper and stopped after lesions of granuloma annulare increased in size. He has had no further PE episodes or seizures but headaches returned after ETN was stopped while still on methotrexate but controlled with over the counter (OTC) medications. Despite cessation of ETN about 3 years earlier and methotrexate about 2.5 years ago, granuloma annulare continues to increase in size on treatment with topical corticosteroids.
2	The patient is a Hispanic male who presented with left sided numbness and weakness, headaches, vision loss and left facial droop due to hydrocephalus and extensive SUBNCC. A shunt was inserted and he was begun on high dose prednisone, albendazole and anti-seizure medications and referred to NIH. At that time, he was started on high dose dexamethasone, along with methotrexate, albendazole and praziquantel. After successfully tapering dexamethasone to about half the starting dose, he mistakenly precipitously lowered the dexamethasone dose he was scheduled to take and became symptomatic. He responded to increased dexamethasone dosing but developed new symptoms of a right sided headache, dizziness and falling to the right, which prompted initiating 50 mg/wk. ETN and a further increase in the dose of dexamethasone. He rapidly became asymptomatic but a month later was unable to return to clinic and lost to follow up because he was able to find work and could not take any time off. Altogether he was under our care for about 2.5 months and took ETN for 31 days.
3	A patient with multiple manifestations of NCC including ventricular, parenchymal and possibly resolved SUBNCC. Following successful treatment of parenchymal disease and shunt placements for hydrocephalus and lateral ventricular entrapment, the patient began to experience seizures, headaches, irritability and inattention as a result of recurrent PE episodes involving multiple large, enhancing calcifications. After developing a pattern of spontaneous repeated PE episodes, ETN was initiated after 4 calcifications developed PE at the same time, associated with irritability, inattention and headaches (HAs). 5 weeks later, the patient was greatly improved; ETN was maintained for the next 10 months. 14 months later ETN was restarted after she developed new PE episodes involving 4 different calcifications. The patient stopped ETN 5 months later when she misinterpreted arm pain from a cervical disc for an ETN side effect and stopped the medication. After stopping her last course of ETN almost a year ago, she remains asymptomatic without additional PE episodes, HAs or seizures.
4	After migrating to the U.S. from India 18 years prior to diagnosis, the patient developed signs and symptoms of spinal arachnoiditis. Despite many evaluations including several internationally recognized medical institutions, she remained undiagnosed for 15 years

	until the fall of 2017. She had been recently started on anakinra (a human interleukin 1 receptor antagonist protein) elsewhere to control inflammation due to a process of unclear cause with some improvement in subjective symptoms. She was able to tolerate a decrease in prednisone from about 35 mg/day to 29 mg/day, but despite multiple attempts she has been unable to taper prednisone any lower. Following the diagnosis of NCC, she was started on 50 mg/day of ETN, albendazole and praziquantel therapy; anakinra was continued because the patient was adamant it had controlled some of her symptoms. Subsequently, after starting on ETN in the absence of other changes in medications, her activity level increased and she was now able to gradually lower her daily dose of prednisone, which currently stands at 0.5 mg/day.
5	A 19 y/o Hispanic female initially presented with focal seizures manifested as visual changes, HAs, nausea and vomiting due to a single degenerating cyst in the left occipital lobe. Treatment with albendazole and corticosteroids was complicated by corticosteroid induced psychosis requiring hospitalization. One year later, after the cyst had calcified, she presented with flashing lights and unrelenting HAs due recurring PE episodes involving the single calcification. She was started on ETN without other immunosuppressive medications with clear but partial improvement and subsequent worsening upon ETN withdrawal. In total she was given 3 courses of ETN for recurrent symptoms. After almost a year, the patient remains asymptomatic without further PE episodes on anti-seizure medications.
6	A patient with complex SUBNCC disease complicated by eosinophilic meningitis, hydrocephalus and significant long tract signs and symptoms was begun on albendazole, praziquantel, corticosteroids, methotrexate, ETN and treatment for latent tuberculosis while hospitalized. Shortly after discharge in the midst of a corticosteroid taper, she redeveloped multiple long tract signs and symptoms necessitating simplification of her treatment regimen including stopping ETN. She then developed symptomatic hydrocephalus requiring shunt placement. Thereafter, the patient did well on continued treatment resulting in apparent cure. However, on follow up, she was found to have an asymptomatic relapse requiring successful retreatment. She remains well without relapse.
7	The patient presented with a severe headache due to a giant left Sylvian fissure cyst resulting in sizeable mass effect, which was surgically extracted. Although not causing symptoms at the time, there was moderate involvement of all the basilar cisterns. After suboptimal and partial treatments, a few months later there was a massive increase in size and extent of his basilar SUBNCC disease resulting in hydrocephalus. Attempts to treat the patient over the next 3 years were complicated by non-compliance because the patient could not afford cysticidal drugs. He had periods of improvement and worsening depending on drug availability and multiple hospitalized treatments. After being lost to follow up for about 4 years, the patient again presented with now massive compensated hydrocephalus, multiple neurological signs including inability to walk and dementia. There was a significant decrease in size and partial resolution of most of the massive basilar disease. After more aggressive and persistent treatment, he improved and referred to NIH. On reinitiating corticosteroids and continuation of treatment with albendazole and praziquantel, he developed acute corticosteroid induced psychosis requiring hospitalization. Treatment included a fast corticosteroid taper, anti-psychotic

	medication and initiation of ETN treatment as the single immunosuppressive agent, while on combined cysticidal treatment. Despite the continued presence of non-shunted massive compensated hydrocephalus, the patient dramatically improved with residual but significantly improved cognition and the presence of mild neurological impairments. He regained the ability to care for himself, take his own medications correctly, perform menial tasks and gain employment. CSF parameters have almost entirely normalized in parallel with along his clinical improvement. He is stable on no medication and without relapse 4 months after stopping ETN.
8	The patient presented with severe SUBNCC of the brain and spinal cord resulting in hydrocephalus. His initial symptoms and signs were primarily related to brain involvement. He responded well to NCC treatments at the referring university with improvement and stabilization of imaging of spine and brain. However, while brain related symptoms subsided, those caused by spinal disease including severe back pain, dysesthesia of the left leg, and right heel pain worsened and became his major complaints. At NIH he was begun on INH treatment for latent tuberculosis, and subsequently started on ETN in December 2017. Thereafter, he had significant improvement in his back pain and increased mobility in part due to decrease in his back pain. He has almost completed the taper of corticosteroids and continues on ETN treatment.
9	Despite multiple evaluations and hospitalizations for symptoms typical of NCC, the patient remained undiagnosed for 3 years, which allowed significant progression of NCC including severe and extensive SUBNCC, ventricular and spinal disease resulting in a myriad of associated symptoms and signs. Her treatment course was complicated by an increase in symptoms associated with cysticidal treatment, successfully treated with an increase in corticosteroid dosing but an exacerbation of symptoms during subsequent corticosteroid tapers. Albendazole was stopped after she developed reversible agranulocytosis; treatment was continued with praziquantel alone. During corticosteroid taper attempts, most of her reappearing symptoms were attributable to ventriculitis and periventricular edema of the 4th ventricle and resulting in hydrocephalus that responded to an increase in the dose of corticosteroids and cystectomy. Despite 25 mg/kg ETN started early in her treatment course, symptoms reoccurred during renewed corticosteroid tapers. Increasing the dose of ETN to 50 mg/wk. was followed by a successful taper off corticosteroids and all treatments were stopped. Although she subsequently experienced two episodes of shunt malfunction of unclear cause, the patient has done well. Overall the patient has greatly improved with no obvious reoccurrence over 1 year after stopping cysticidal treatment and more than 2 years after stopping ETN. However, the patient still complains of residual chronic symptoms including mild dizziness.
10	The patient was referred for treatment with complicated NCC including extensive basilar SUBNCC and Sylvian fissure involvement, a lacunar infarct, an inoperable basilar artery aneurysm, and mild hydrocephalus. He was started on ETN after he developed worsening hydrocephalus and increased enhancement during a corticosteroid taper while on methotrexate. The taper was successfully completed without worsening hydrocephalus although a small increase in corticosteroid dose and subsequent taper was needed. Because CSF parameters suggested incomplete treatment, he received a second course of treatment. The MRI has been stable and the patient asymptomatic over 2.5 yrs. post

	stopping all treatments. The CSF antigen decreased to non-detectable level but the CSF WBC count remains increased but stable with a high protein value, and normal glucose.
11	The patient was referred for continued treatment of extensive SUBNCC NCC, parenchymal cysts and partial aqueduct of Sylvius obstruction. He had been evaluated and retreated multiple times over 3 years before being seen at NIH and had developed serious corticosteroid side effects. At NIH he was begun on albendazole, praziquantel, methotrexate and corticosteroids. After he developed dizziness and headache during a corticosteroid taper, ETN was added. The patient promptly improved and he quickly decreased and then stopped corticosteroids. He transiently developed atypical headaches responding to standard treatment for migraine headaches. Other medications were stopped shortly thereafter and the patient has done well without relapse or symptoms over the next 5 years.
12	The patient had a 5-year history of SUBNCC disease, several subarachnoid hemorrhages, seizures, spinal disease, severe corticosteroid side effects and multiple complications before being referred to NIH for shunt placement and continued treatment. He was restarted on albendazole, corticosteroids, praziquantel and started on methotrexate. After multiple attempts, he could not be successfully weaned from corticosteroids and had developed symptomatic avascular necrosis of right hip. Following the addition of ETN, corticosteroids were successfully tapered. He was maintained on ETN and methotrexate for another 10 months until he developed shunt failure resulting in acute hydrocephalus. After shunt repair/replacement, corticosteroids and ETN were restarted. The corticosteroids were subsequently tapered and stopped about 3 weeks later; ETN was continued alone for another 5 months. The patient has done well without recurrence over the subsequent 5 years.
13	The patient has an unusual history of SUBNCC including multiple lacunar infarcts and a major large vessel stroke. He was born in the U.S. with minimal exposure to endemic regions during summer cruise vacations and limited shore excursions. Prior to diagnosis, he experienced unexplained transient neurological signs and symptoms accompanied by lymphocytic pleocytosis 13 and 9 years before presentation. Evaluations failed to reveal a cause including MRI studies that were normal. He improved spontaneously. The diagnosis was eventually established by brain biopsy after he presented with symptoms due to multiple lacunar infarcts in the setting of CSF pleocytosis. He responded well to cysticidal and corticosteroid treatments with normalization of CSF parameters including undetectable cestode CSF antigen but nevertheless experienced a major infarct of the posterior inferior cerebellar artery associated with a dramatic increase in CSF pleocytosis while off of all drugs. Cestode antigen values were not measured at the time of the stroke but were undetectable before the stroke and a month after the stroke. He was restarted on cysticidal medication, corticosteroids, methotrexate and begun on ETN . Presumably the infarction occurred as a result of residual inflammation originating from degenerating residual parasite. He is stable over 3 years later in the absence of recurring disease and near normal CSF parameters including non-detectable cestode antigen, but suffers from disabling sequelae due to his prior infarcts.
14	The patient became acutely ill due to a ventricular cyst likely traversing the aqueduct of Sylvius into the 4th ventricle. He experienced episodes of hydrocephalus and multiple shunt revisions and was eventually diagnosed with a shunt infection. After shunt

	replacement, he developed severe ventriculitis and periventricular edema around the retained 4th ventricular cyst prompting cysticidal medications along with methotrexate, and high dose steroids. He had also been earlier started on INH for latent TB. LFT abnormalities developed and methotrexate, albendazole and INH were stopped with continuation of corticosteroids and praziquantel. Because of persistence of ventriculitis and periventricular edema, ETN was initiated and corticosteroids were subsequently tapered off. The patient slowly became asymptomatic. ETN was continued for another year with resolution of the ventriculitis, periventricular edema and subsequent complete recovery. He remains asymptomatic over 4.5 years after stopping all treatments.
15	The patient had a 20-year history of neurocysticercosis initially diagnosed at the time she was treated for a pituitary adenoma. Although growth was documented and medication prescribed, the patient never took the medication. She eventually presented with massive SUBNCC disease. Her major symptoms were headaches and hemiparalytic episodes. She was treated with cysticidal medication and corticosteroids with improvement. Methotrexate was added to control symptoms of headaches and dizziness. Nevertheless, because of the development of acute left sided hemiparesis without any changes in the MRI imaging, she was restarted on high dose steroids and ETN was added to methotrexate. She clinically improved with a marked decrease in headaches and ETN was stopped. However, after cessation of ETN, headaches and dizziness reoccurred although they were not as severe. Methotrexate was then stopped. After cessation of immunosuppressive medications, she experienced another transient paralysis episode involving both sides of her body, which again resolved spontaneously. The pathophysiology of the left sided symptoms remains unclear and the MRI has remained unchanged without correlation to her clinical state. Her headaches and dizziness have improved overall and allowed her to contribute to house-keeping chores with the help of OTC medications.
16	The patient is a 47 y/o Hispanic male with a degenerating cyst in the right motor strip controlling the left side of the tongue. The lesion developed partial and then increasing calcification over time. He also has multiple other parenchymal calcifications of no clinical significance. Multiple PE episodes occurred around the lesion, resulting in focal and generalized seizures. Symptoms, which were sometimes prolonged and continuous, involved the left side of the tongue and consisted of tonic contractions of the left side of the tongue induced by eating and drinking as well as extremely bothersome dysesthesias. Symptoms also included intermittent numbness weakness of the left arm and occasionally the left leg. Although partially controlled with high dose corticosteroids, symptoms returned when corticosteroids were tapered. Upon referral to NIH, the patient was started on ETN. However, because the patient developed another PE episode a few weeks after initiation of ETN, he was readmitted to NIH for reevaluation and change of therapy. Unexpectedly, the patient refused to stop taking ETN insisting that the drug was helping him because the dysesthesias had abated. On continued ETN and antiseizure medications, corticosteroids were successfully tapered and the patient clinically improved with decreased seizure activity and he regained his ability to work and eat. He is stable off of ETN and corticosteroids. The lesion's size and enhancement considerably decreased over this time frame.
